# 3-(4-Chloro­phen­yl)-5-(4-eth­oxy­phen­yl)-4,5-dihydro-1*H*-pyrazole-1-carbothio­amide ethanol monosolvate

**DOI:** 10.1107/S1600536813005369

**Published:** 2013-03-02

**Authors:** Ching Kheng Quah, Hoong-Kun Fun, Thitipone Suwunwong, Nawong Boonnak, Suchada Chantrapromma

**Affiliations:** a X-ray Crystallography Unit, School of Physics, Universiti Sains Malaysia, 11800 USM, Penang, Malaysia; b Department of Pharmaceutical Chemistry, College of Pharmacy, King Saud University, PO Box 2457, Riyadh 11451, Saudi Arabia; c Department of Chemistry, Faculty of Science, Prince of Songkla University, Hat-Yai, Songkhla 90112, Thailand; d Faculty of Traditional Thai Medicine, Prince of Songkla University, Hat-Yai, Songkhla 90112, Thailand

## Abstract

The asymmetric unit of the title compound, C_18_H_18_ClN_3_OS·C_2_H_5_OH, comprises a pyrazoline derivative and an ethanol solvent mol­ecule. In the mol­ecule of the pyrazoline derivative, the pyrazole ring adopts an envelope conformation with the C atom bearing the eth­oxy­phenyl substituent as the flap. The dihedral angle between the benzene rings is 74.22 (7)°. The eth­oxy group is coplanar with the attached benzene ring [C—O—C—C_meth­yl_ = 175.50 (11)° and r.m.s. deviation = 0.0459 (1) Å for the nine non-H atoms]. In the crystal, the pyrazoline mol­ecules are linked by N—H⋯O_eth­oxy_ hydrogen bonds into chains along the *c* axis and are further linked with the solvent ethanol mol­ecules by N—H⋯O_ethanol_ and O_ethanol_—H⋯S hydrogen bonds. C—H⋯π inter­actions are also present.

## Related literature

For bond-length data, see: Allen *et al.* (1987 ▶). For ring conformational analysis, see: Cremer & Pople (1975 ▶). For related structures, see: Chantrapromma *et al.* (2012 ▶); Nonthason *et al.* (2011 ▶). For background to and applications of pyrazoline derivatives, see: Bilgin *et al.* (1992 ▶, 1993 ▶, 1994 ▶); Gokhan *et al.* (2003 ▶); Ruhoglu *et al.* (2005 ▶); Zhang *et al.* (2000 ▶). For the fluorescent properties and anti­oxidant activity of pyrazoline derivatives by DPPH scavenging, see: Molyneux (2004 ▶). For the stability of the temperature controller used in the data collection, see: Cosier & Glazer (1986 ▶).
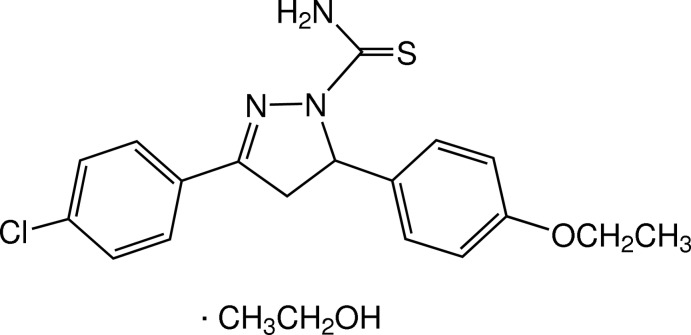


## Experimental

### 

#### Crystal data

C_18_H_18_ClN_3_OS·C_2_H_6_O
*M* *_r_* = 405.94
Monoclinic, 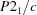

*a* = 9.3145 (4) Å
*b* = 25.3673 (12) Å
*c* = 9.5565 (5) Å
β = 115.082 (1)°
*V* = 2045.12 (17) Å^3^
*Z* = 4
Mo *K*α radiation
μ = 0.31 mm^−1^
*T* = 100 K
0.49 × 0.24 × 0.24 mm

#### Data collection

Bruker APEXII CCD area-detector diffractometer
Absorption correction: multi-scan (*SADABS*; Bruker, 2005 ▶) *T*_min_ = 0.864, *T*_max_ = 0.929
17996 measured reflections
3514 independent reflections
3228 reflections with *I* > 2σ(*I*)
*R*_int_ = 0.023

#### Refinement

*R*[*F* ^2^ > 2σ(*F* ^2^)] = 0.027
*wR*(*F* ^2^) = 0.071
*S* = 1.06
3514 reflections
258 parameters
H atoms treated by a mixture of independent and constrained refinement
Δρ_max_ = 0.29 e Å^−3^
Δρ_min_ = −0.21 e Å^−3^

### 

Data collection: *APEX2* (Bruker, 2005 ▶); cell refinement: *SAINT* (Bruker, 2005 ▶); data reduction: *SAINT*; program(s) used to solve structure: *SHELXTL* (Sheldrick, 2008 ▶); program(s) used to refine structure: *SHELXTL*; molecular graphics: *SHELXTL*; software used to prepare material for publication: *SHELXTL*, *PLATON* (Spek, 2009 ▶) and *publCIF* (Westrip, 2010 ▶).

## Supplementary Material

Crystal structure: contains datablock(s) global, I. DOI: 10.1107/S1600536813005369/rz5045sup1.cif

Structure factors: contains datablock(s) I. DOI: 10.1107/S1600536813005369/rz5045Isup2.hkl

Supplementary material file. DOI: 10.1107/S1600536813005369/rz5045Isup3.cml

Additional supplementary materials:  crystallographic information; 3D view; checkCIF report

## Figures and Tables

**Table 1:** Hydrogen-bond geometry (Å, °) *Cg*1 and *Cg*2 are the centroids of the C1–C6 and C10–C15 rings, respectively.

*D*—H⋯*A*	*D*—H	H⋯*A*	*D*⋯*A*	*D*—H⋯*A*
N3—H2*N*3⋯O1^i^	0.879 (18)	2.225 (18)	3.0531 (16)	157.1 (17)
N3—H1*N*3⋯O2^i^	0.857 (19)	2.019 (19)	2.8324 (18)	158.0 (16)
O2—H1*O*2⋯S2^ii^	0.85 (2)	2.43 (2)	3.2340 (12)	159.1 (16)
C5—H5*A*⋯*Cg*2^iii^	0.95	2.96	3.5701 (15)	123
C8—H8*A*⋯*Cg*1^iv^	0.99	2.89	3.8543 (17)	165

Symmetry codes: (i) 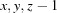; (ii) 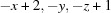; (iii) 
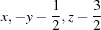
; (iv) 
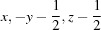
.

**Fig. 1.**
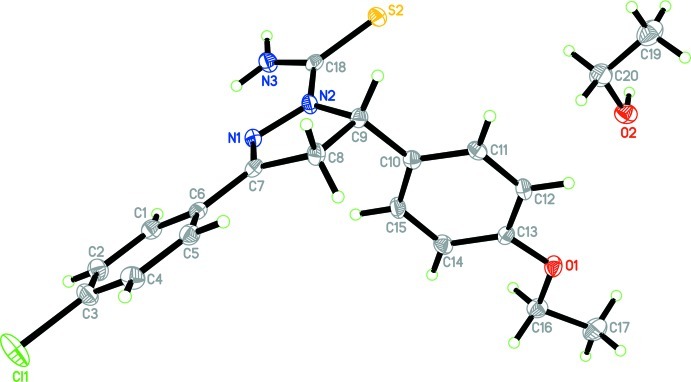
The molecular structure of the title compound, showing 50% probability displacement ellipsoids and the atom-numbering scheme.

**Fig. 2.**
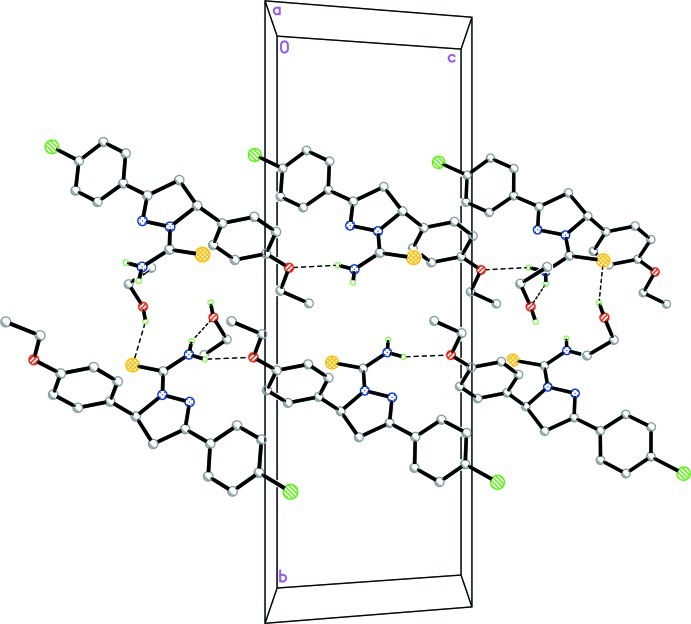
The crystal packing of the title compound viewed along the a axis. Only H atoms involved in hydrogen bonding were shown for the sake of clarity. Hydrogen bonds were drawn as dashed lines.

**Fig. 3.**
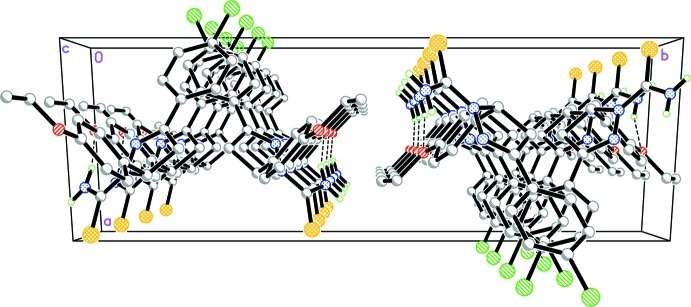
The crystal packing of the the title compound viewed along the c axis, showing the stacking of pyrazoline derivatives. Solvent ethanol molecules are omitted and for the sake of clarity, only H atoms involved in hydrogen bonding are shown. Hydrogen bonds were drawn as dashed lines.

## supplementary crystallographic information

### Comment

The studies of pyrazoline derivatives have gained increasing interests in the therapeutic research field due to their broad spectrum of biological properties such as antihypotensive, muscle relaxant, anticonvulsant, psychoanaleptic and antidepressant activities (Bilgin *et al.*, 1992; Bilgin *et al.*, 1993; Bilgin *et al.*, 1994; Gokhan *et al.*, 2003; Ruhoglu *et al.*, 2005). The previous studies by Bilgin and co-workers (Bilgin *et al.*, 1993) reported the synthesis of thiocarbamoyl pyrazoline derivatives which exhibited significant antidepressant activity. Moreover pyrazoline derivatives with the phenyl group at the 5-position have been shown to exhibit excellent blue photoluminescence (Zhang *et al.*, 2000). In view of the importance of pyrazoline derivatives, we have synthesized a series of thiocarbamoyl pyrazoline derivatives by the cyclization reaction between chalcones and thiosemicarbazide and have studied their fluorescence, antioxidant and antityrosinase activities. The title compound (I) was synthesized and evaluated for fluorescent property and antioxidant activity by DPPH scavenging (Molyneux, 2004). Unfortunately our results show that (I) did not exhibit fluorescence and was found to be inactive for antioxidant and antityrosinase activities. Herein we report the synthesis and crystal structure of (I).

In the molecule of the title ethanol monosolvated pyrazoline derivative (Fig. 1), C_18_H_18_ClN_3_OS.C_2_H_5_OH, the pyrazole ring adopts an envelope conformation with the slightly puckered C9 atom having the maximum deviation of -0.0950 (15) Å, and the puckering parameter Q = 0.1508 (15) Å and φ = 257.2 (5)° (Cremer & Pople, 1975). The mean plane through the pyrazole ring forms dihedral angles of 15.82 (8) and 82.29 (8)° with the chloro-substituted and ethoxy-substituted rings, respectively, whereas the dihedral angle between the chloro-substituted and ethoxy-substituted rings is 74.22 (7)°. The conformation of the carbothioamide unit with respect to the pyrazole ring can be indicated by the torsion angles N1–N2–C18–N3 = -6.07 (18)° and N1–N2–C18–S2 = 173.50 (9)°. The ethoxy group lies nearly on the same plane of the attached benzene ring with the torsion angle C13–O1–C16–C17 = 175.50 (11)°, and r.m.s. of 0.0459 (1) Å for the nine non H atoms (C10, C11, C12, C13, C14, C15, O1, C16 and C17). Bond distances of (I) are in normal range (Allen *et al.*, 1987) and comparable with those observed in related structures (Chantrapromma *et al.*, 2012; Nonthason *et al.*, 2011)

In the crystal packing (Fig.2), the pyrazoline molecules are linked together by N—H···O_ethoxy_ hydrogen bonds (Table 1) into chains along the [0 0 1] direction and are further linked with the solvent ethanol molecules by N—H···O_ethanol_ and O_ethanol_—H···S hydrogen bonds (Table 1). C—H···π interactions were also presented (Table 1). Fig. 3 shows the stacking of pyrazoline molecules along the *c* axis.

### Experimental

The title compound was synthesized by dissolving (*E*)-1-(4-chlorophenyl)-3-(4-ethoxyphenyl)prop-2-en-1-one (0.29 g, 1 mmol) in ethanol (10 ml) and the solution of an excess thiosemicarbazide (0.18 g, 2 mmol) in a solution of KOH (0.11 g, 2 mmol) in ethanol (15 ml) was then added. The reaction mixture was vigorously stirred and refluxed for 4 h. The pale yellow solid of the title compound was obtained after cooling of the reaction and was then filtered off under vacuum. Pale yellow block-shaped single crystals of the title compound suitable for *X*-ray structure determination were recrystallized from CH_3_OH/C_2_H_5_OH (1:1 *v*/*v*) by slow evaporation of the solvent at room temperature after several days. M. p.: 406–407 K.

### Refinement

Amide and ethanol H atoms were located from difference Fourier maps and refined isotropically. The remaining H atoms were positioned geometrically and allowed to ride on their parent atoms, with d(C-H) = 0.95 Å for aromatic, 1.00 Å for CH, 0.99 Å for CH_2_ and 0.98 Å for CH_3_ atoms. The *U*_iso_ values were constrained to be 1.5*U*_eq_ of the carrier atom for methyl H atoms and 1.2*U*_eq_ for the remaining H atoms. A rotating group model was used for the methyl groups.

### Crystal data

**Table:**

C_18_H_18_ClN_3_OS·C_2_H_6_O	*F*(000) = 856
*M**_r_* = 405.94	*D*_x_ = 1.318 Mg m^−^^3^
Monoclinic, *P*2_1_/*c*	Melting point = 406–407 K
Hall symbol: -P 2ybc	Mo *K*α radiation, λ = 0.71073 Å
*a* = 9.3145 (4) Å	Cell parameters from 3514 reflections
*b* = 25.3673 (12) Å	θ = 1.6–25.0°
*c* = 9.5565 (5) Å	µ = 0.31 mm^−^^1^
β = 115.082 (1)°	*T* = 100 K
*V* = 2045.12 (17) Å^3^	Block, pale yellow
*Z* = 4	0.49 × 0.24 × 0.24 mm

### Data collection

**Table:**

Bruker APEXII CCD area-detector diffractometer	3514 independent reflections
Radiation source: sealed tube	3228 reflections with *I* > 2σ(*I*)
Graphite monochromator	*R*_int_ = 0.023
φ and ω scans	θ_max_ = 25.0°, θ_min_ = 1.6°
Absorption correction: multi-scan (*SADABS*; Bruker, 2005)	*h* = −11→10
*T*_min_ = 0.864, *T*_max_ = 0.929	*k* = −30→30
17996 measured reflections	*l* = −11→11

### Refinement

**Table:**

Refinement on *F*^2^	Primary atom site location: structure-invariant direct methods
Least-squares matrix: full	Secondary atom site location: difference Fourier map
*R*[*F*^2^ > 2σ(*F*^2^)] = 0.027	Hydrogen site location: inferred from neighbouring sites
*wR*(*F*^2^) = 0.071	H atoms treated by a mixture of independent and constrained refinement
*S* = 1.06	*w* = 1/[σ^2^(*F*_o_^2^) + (0.0305*P*)^2^ + 1.0598*P*] where *P* = (*F*_o_^2^ + 2*F*_c_^2^)/3
3514 reflections	(Δ/σ)_max_ = 0.001
258 parameters	Δρ_max_ = 0.29 e Å^−^^3^
0 restraints	Δρ_min_ = −0.21 e Å^−^^3^

### Special details

**Table:**

Experimental. The crystal was placed in the cold stream of an Oxford Cryosystems Cobra open-flow nitrogen cryostat (Cosier & Glazer, 1986) operating at 120.0 (1) K.
Geometry. All esds (except the esd in the dihedral angle between two l.s. planes) are estimated using the full covariance matrix. The cell esds are taken into account individually in the estimation of esds in distances, angles and torsion angles; correlations between esds in cell parameters are only used when they are defined by crystal symmetry. An approximate (isotropic) treatment of cell esds is used for estimating esds involving l.s. planes.
Refinement. Refinement of F^2^ against ALL reflections. The weighted R-factor wR and goodness of fit S are based on F^2^, conventional R-factors R are based on F, with F set to zero for negative F^2^. The threshold expression of F^2^ > 2sigma(F^2^) is used only for calculating R-factors(gt) etc. and is not relevant to the choice of reflections for refinement. R-factors based on F^2^ are statistically about twice as large as those based on F, and R- factors based on ALL data will be even larger.

### Fractional atomic coordinates and isotropic or equivalent isotropic displacement parameters (Å^2^)

**Table:**

	*x*	*y*	*z*	*U*_iso_*/*U*_eq_	
Cl1	−0.11105 (4)	0.286557 (14)	−0.62261 (5)	0.03191 (12)	
S2	0.90677 (4)	0.091862 (13)	0.20892 (4)	0.01689 (10)	
O1	0.45072 (11)	0.07649 (4)	0.58383 (11)	0.0186 (2)	
O2	0.89420 (12)	0.00951 (4)	0.81489 (12)	0.0234 (2)	
N1	0.51075 (13)	0.15074 (4)	−0.10757 (13)	0.0156 (2)	
N2	0.63750 (13)	0.13984 (4)	0.03376 (12)	0.0153 (2)	
N3	0.70567 (15)	0.07278 (5)	−0.08430 (14)	0.0188 (3)	
C1	0.22275 (15)	0.18609 (5)	−0.35513 (16)	0.0170 (3)	
H1A	0.2529	0.1506	−0.3600	0.020*	
C2	0.09364 (16)	0.20717 (5)	−0.47764 (16)	0.0191 (3)	
H2A	0.0343	0.1864	−0.5663	0.023*	
C3	0.05218 (16)	0.25928 (6)	−0.46868 (16)	0.0198 (3)	
C4	0.13604 (16)	0.29046 (5)	−0.34122 (17)	0.0194 (3)	
H4A	0.1060	0.3260	−0.3377	0.023*	
C5	0.26506 (16)	0.26870 (5)	−0.21847 (16)	0.0167 (3)	
H5A	0.3235	0.2896	−0.1300	0.020*	
C6	0.30981 (15)	0.21648 (5)	−0.22366 (15)	0.0152 (3)	
C7	0.44749 (15)	0.19435 (5)	−0.09361 (15)	0.0145 (3)	
C8	0.53006 (16)	0.21960 (5)	0.06381 (15)	0.0166 (3)	
H8A	0.4538	0.2289	0.1071	0.020*	
H8B	0.5888	0.2516	0.0595	0.020*	
C9	0.64433 (15)	0.17545 (5)	0.15886 (15)	0.0150 (3)	
H9A	0.7540	0.1898	0.2160	0.018*	
C10	0.59077 (15)	0.14822 (5)	0.26960 (15)	0.0144 (3)	
C11	0.66466 (15)	0.15890 (5)	0.42750 (15)	0.0157 (3)	
H11A	0.7516	0.1827	0.4662	0.019*	
C12	0.61322 (15)	0.13533 (5)	0.52878 (15)	0.0163 (3)	
H12A	0.6637	0.1434	0.6358	0.020*	
C13	0.48746 (15)	0.09970 (5)	0.47389 (15)	0.0149 (3)	
C14	0.41061 (16)	0.08924 (5)	0.31602 (16)	0.0183 (3)	
H14A	0.3231	0.0657	0.2770	0.022*	
C15	0.46348 (16)	0.11366 (5)	0.21675 (15)	0.0182 (3)	
H15A	0.4109	0.1065	0.1092	0.022*	
C16	0.32760 (16)	0.03716 (6)	0.53447 (16)	0.0204 (3)	
H16A	0.3520	0.0088	0.4768	0.024*	
H16B	0.2245	0.0531	0.4663	0.024*	
C17	0.32115 (19)	0.01539 (6)	0.67807 (19)	0.0280 (3)	
H17A	0.2386	−0.0117	0.6495	0.042*	
H17B	0.2967	0.0439	0.7338	0.042*	
H17C	0.4239	−0.0002	0.7445	0.042*	
C18	0.74149 (15)	0.10126 (5)	0.04269 (15)	0.0149 (3)	
C19	1.0976 (2)	0.07701 (7)	0.8804 (2)	0.0324 (4)	
H19A	1.1349	0.1057	0.8353	0.049*	
H19B	1.1843	0.0520	0.9323	0.049*	
H19C	1.0623	0.0916	0.9555	0.049*	
C20	0.96096 (18)	0.04889 (6)	0.75385 (18)	0.0272 (3)	
H20A	0.8781	0.0749	0.6948	0.033*	
H20B	0.9987	0.0322	0.6818	0.033*	
H2N3	0.620 (2)	0.0803 (7)	−0.168 (2)	0.025 (4)*	
H1N3	0.772 (2)	0.0504 (7)	−0.0899 (19)	0.023 (4)*	
H1O2	0.957 (2)	−0.0164 (8)	0.835 (2)	0.044 (6)*	

### Atomic displacement parameters (Å^2^)

**Table:**

	*U*^11^	*U*^22^	*U*^33^	*U*^12^	*U*^13^	*U*^23^
Cl1	0.0265 (2)	0.02200 (19)	0.0290 (2)	0.00534 (14)	−0.00589 (16)	0.00216 (15)
S2	0.01535 (17)	0.01703 (18)	0.01616 (18)	0.00211 (12)	0.00463 (14)	0.00103 (12)
O1	0.0217 (5)	0.0193 (5)	0.0151 (5)	−0.0045 (4)	0.0081 (4)	0.0002 (4)
O2	0.0202 (5)	0.0218 (5)	0.0279 (6)	0.0030 (4)	0.0099 (4)	−0.0036 (4)
N1	0.0148 (5)	0.0188 (6)	0.0127 (5)	0.0017 (4)	0.0052 (5)	0.0019 (4)
N2	0.0162 (5)	0.0172 (6)	0.0110 (5)	0.0035 (4)	0.0043 (5)	0.0007 (4)
N3	0.0180 (6)	0.0205 (6)	0.0165 (6)	0.0054 (5)	0.0059 (5)	−0.0017 (5)
C1	0.0186 (7)	0.0153 (6)	0.0190 (7)	0.0011 (5)	0.0099 (6)	0.0002 (5)
C2	0.0187 (7)	0.0199 (7)	0.0169 (7)	−0.0014 (5)	0.0058 (6)	−0.0016 (5)
C3	0.0155 (6)	0.0207 (7)	0.0194 (7)	0.0020 (5)	0.0039 (6)	0.0044 (6)
C4	0.0197 (7)	0.0150 (7)	0.0232 (7)	0.0028 (5)	0.0090 (6)	0.0012 (5)
C5	0.0176 (6)	0.0176 (7)	0.0160 (7)	−0.0005 (5)	0.0080 (6)	−0.0010 (5)
C6	0.0146 (6)	0.0178 (7)	0.0159 (7)	0.0002 (5)	0.0090 (6)	0.0024 (5)
C7	0.0165 (6)	0.0151 (6)	0.0146 (7)	−0.0005 (5)	0.0093 (5)	0.0011 (5)
C8	0.0202 (7)	0.0150 (6)	0.0144 (7)	0.0018 (5)	0.0073 (6)	0.0010 (5)
C9	0.0167 (6)	0.0150 (6)	0.0138 (6)	0.0003 (5)	0.0069 (5)	−0.0011 (5)
C10	0.0154 (6)	0.0128 (6)	0.0145 (6)	0.0036 (5)	0.0057 (5)	0.0008 (5)
C11	0.0141 (6)	0.0142 (6)	0.0167 (7)	−0.0004 (5)	0.0045 (5)	−0.0009 (5)
C12	0.0182 (6)	0.0165 (6)	0.0115 (6)	0.0012 (5)	0.0038 (5)	−0.0006 (5)
C13	0.0174 (6)	0.0139 (6)	0.0147 (7)	0.0032 (5)	0.0083 (5)	0.0023 (5)
C14	0.0170 (7)	0.0189 (7)	0.0171 (7)	−0.0039 (5)	0.0053 (6)	−0.0018 (5)
C15	0.0190 (7)	0.0216 (7)	0.0114 (6)	−0.0016 (5)	0.0038 (6)	−0.0016 (5)
C16	0.0197 (7)	0.0195 (7)	0.0232 (7)	−0.0039 (5)	0.0103 (6)	−0.0007 (6)
C17	0.0324 (8)	0.0263 (8)	0.0321 (9)	−0.0050 (6)	0.0203 (7)	0.0027 (6)
C18	0.0161 (6)	0.0145 (6)	0.0167 (7)	−0.0010 (5)	0.0096 (6)	0.0019 (5)
C19	0.0400 (9)	0.0287 (8)	0.0365 (9)	−0.0080 (7)	0.0240 (8)	−0.0059 (7)
C20	0.0263 (8)	0.0299 (8)	0.0259 (8)	0.0076 (6)	0.0116 (7)	0.0047 (6)

### Geometric parameters (Å, º)

**Table:**

Cl1—C3	1.7488 (14)	C8—H8A	0.9900
S2—C18	1.6962 (13)	C8—H8B	0.9900
O1—C13	1.3685 (16)	C9—C10	1.5145 (19)
O1—C16	1.4402 (16)	C9—H9A	1.0000
O2—C20	1.4256 (19)	C10—C15	1.3863 (19)
O2—H1O2	0.85 (2)	C10—C11	1.3941 (19)
N1—C7	1.2866 (18)	C11—C12	1.385 (2)
N1—N2	1.3938 (15)	C11—H11A	0.9500
N2—C18	1.3539 (17)	C12—C13	1.3939 (19)
N2—C9	1.4782 (17)	C12—H12A	0.9500
N3—C18	1.3276 (18)	C13—C14	1.3941 (19)
N3—H2N3	0.879 (19)	C14—C15	1.387 (2)
N3—H1N3	0.856 (18)	C14—H14A	0.9500
C1—C2	1.3817 (19)	C15—H15A	0.9500
C1—C6	1.4024 (19)	C16—C17	1.504 (2)
C1—H1A	0.9500	C16—H16A	0.9900
C2—C3	1.390 (2)	C16—H16B	0.9900
C2—H2A	0.9500	C17—H17A	0.9800
C3—C4	1.383 (2)	C17—H17B	0.9800
C4—C5	1.3892 (19)	C17—H17C	0.9800
C4—H4A	0.9500	C19—C20	1.512 (2)
C5—C6	1.3958 (19)	C19—H19A	0.9800
C5—H5A	0.9500	C19—H19B	0.9800
C6—C7	1.4673 (18)	C19—H19C	0.9800
C7—C8	1.5112 (18)	C20—H20A	0.9900
C8—C9	1.5468 (18)	C20—H20B	0.9900
			
C13—O1—C16	118.05 (10)	C11—C10—C9	120.66 (12)
C20—O2—H1O2	104.9 (14)	C12—C11—C10	120.95 (12)
C7—N1—N2	107.89 (11)	C12—C11—H11A	119.5
C18—N2—N1	119.61 (11)	C10—C11—H11A	119.5
C18—N2—C9	127.33 (11)	C11—C12—C13	120.10 (12)
N1—N2—C9	113.00 (10)	C11—C12—H12A	119.9
C18—N3—H2N3	119.5 (11)	C13—C12—H12A	119.9
C18—N3—H1N3	120.6 (11)	O1—C13—C12	115.71 (11)
H2N3—N3—H1N3	119.2 (16)	O1—C13—C14	124.62 (12)
C2—C1—C6	120.76 (12)	C12—C13—C14	119.66 (12)
C2—C1—H1A	119.6	C15—C14—C13	119.14 (12)
C6—C1—H1A	119.6	C15—C14—H14A	120.4
C1—C2—C3	118.72 (13)	C13—C14—H14A	120.4
C1—C2—H2A	120.6	C10—C15—C14	122.03 (12)
C3—C2—H2A	120.6	C10—C15—H15A	119.0
C4—C3—C2	122.02 (13)	C14—C15—H15A	119.0
C4—C3—Cl1	118.66 (11)	O1—C16—C17	106.85 (11)
C2—C3—Cl1	119.32 (11)	O1—C16—H16A	110.4
C3—C4—C5	118.68 (13)	C17—C16—H16A	110.4
C3—C4—H4A	120.7	O1—C16—H16B	110.4
C5—C4—H4A	120.7	C17—C16—H16B	110.4
C4—C5—C6	120.77 (13)	H16A—C16—H16B	108.6
C4—C5—H5A	119.6	C16—C17—H17A	109.5
C6—C5—H5A	119.6	C16—C17—H17B	109.5
C5—C6—C1	119.05 (12)	H17A—C17—H17B	109.5
C5—C6—C7	119.99 (12)	C16—C17—H17C	109.5
C1—C6—C7	120.95 (12)	H17A—C17—H17C	109.5
N1—C7—C6	121.02 (12)	H17B—C17—H17C	109.5
N1—C7—C8	113.92 (11)	N3—C18—N2	116.09 (12)
C6—C7—C8	125.05 (11)	N3—C18—S2	123.80 (11)
C7—C8—C9	102.17 (10)	N2—C18—S2	120.11 (10)
C7—C8—H8A	111.3	C20—C19—H19A	109.5
C9—C8—H8A	111.3	C20—C19—H19B	109.5
C7—C8—H8B	111.3	H19A—C19—H19B	109.5
C9—C8—H8B	111.3	C20—C19—H19C	109.5
H8A—C8—H8B	109.2	H19A—C19—H19C	109.5
N2—C9—C10	111.87 (10)	H19B—C19—H19C	109.5
N2—C9—C8	100.63 (10)	O2—C20—C19	111.65 (13)
C10—C9—C8	113.08 (11)	O2—C20—H20A	109.3
N2—C9—H9A	110.3	C19—C20—H20A	109.3
C10—C9—H9A	110.3	O2—C20—H20B	109.3
C8—C9—H9A	110.3	C19—C20—H20B	109.3
C15—C10—C11	118.09 (12)	H20A—C20—H20B	108.0
C15—C10—C9	121.20 (12)		
			
C7—N1—N2—C18	−168.23 (12)	C7—C8—C9—N2	13.98 (12)
C7—N1—N2—C9	9.16 (14)	C7—C8—C9—C10	−105.49 (12)
C6—C1—C2—C3	0.5 (2)	N2—C9—C10—C15	−39.66 (16)
C1—C2—C3—C4	0.0 (2)	C8—C9—C10—C15	73.12 (15)
C1—C2—C3—Cl1	−179.93 (11)	N2—C9—C10—C11	142.87 (12)
C2—C3—C4—C5	−0.3 (2)	C8—C9—C10—C11	−104.34 (14)
Cl1—C3—C4—C5	179.57 (11)	C15—C10—C11—C12	0.53 (19)
C3—C4—C5—C6	0.3 (2)	C9—C10—C11—C12	178.07 (12)
C4—C5—C6—C1	0.1 (2)	C10—C11—C12—C13	1.0 (2)
C4—C5—C6—C7	179.33 (12)	C16—O1—C13—C12	−176.59 (11)
C2—C1—C6—C5	−0.5 (2)	C16—O1—C13—C14	2.15 (19)
C2—C1—C6—C7	−179.69 (12)	C11—C12—C13—O1	176.78 (11)
N2—N1—C7—C6	−179.82 (11)	C11—C12—C13—C14	−2.0 (2)
N2—N1—C7—C8	1.51 (15)	O1—C13—C14—C15	−177.19 (12)
C5—C6—C7—N1	−165.56 (13)	C12—C13—C14—C15	1.5 (2)
C1—C6—C7—N1	13.6 (2)	C11—C10—C15—C14	−1.1 (2)
C5—C6—C7—C8	13.0 (2)	C9—C10—C15—C14	−178.60 (12)
C1—C6—C7—C8	−167.88 (13)	C13—C14—C15—C10	0.1 (2)
N1—C7—C8—C9	−10.54 (15)	C13—O1—C16—C17	175.50 (11)
C6—C7—C8—C9	170.85 (12)	N1—N2—C18—N3	−6.07 (18)
C18—N2—C9—C10	−77.42 (16)	C9—N2—C18—N3	176.96 (12)
N1—N2—C9—C10	105.44 (12)	N1—N2—C18—S2	173.50 (9)
C18—N2—C9—C8	162.24 (12)	C9—N2—C18—S2	−3.47 (18)
N1—N2—C9—C8	−14.90 (13)		

### Hydrogen-bond geometry (Å, º)

*Cg*1 and *Cg*2 are the centroids of the C1–C6 and C10–C15 rings, respectively.

**Table:**

*D*—H···*A*	*D*—H	H···*A*	*D*···*A*	*D*—H···*A*
N3—H2*N*3···O1^i^	0.879 (18)	2.225 (18)	3.0531 (16)	157.1 (17)
N3—H1*N*3···O2^i^	0.857 (19)	2.019 (19)	2.8324 (18)	158.0 (16)
O2—H1*O*2···S2^ii^	0.85 (2)	2.43 (2)	3.2340 (12)	159.1 (16)
C5—H5*A*···*Cg*2^iii^	0.95	2.96	3.5701 (15)	123
C8—H8*A*···*Cg*1^iv^	0.99	2.89	3.8543 (17)	165

Symmetry codes: (i) *x*, *y*, *z*−1; (ii) −*x*+2, −*y*, −*z*+1; (iii) *x*, −*y*−1/2, *z*−3/2; (iv) *x*, −*y*−1/2, *z*−1/2.
